# Complete chloroplast genomes of medicinally important *Teucrium* species and comparative analyses with related species from Lamiaceae

**DOI:** 10.7717/peerj.7260

**Published:** 2019-07-09

**Authors:** Arif Khan, Sajjad Asaf, Abdul Latif Khan, Adil Khan, Ahmed Al-Harrasi, Omar Al-Sudairy, Noor Mazin AbdulKareem, Nadiya Al-Saady, Ahmed Al-Rawahi

**Affiliations:** 1 Natural and Medical Sciences Research Center, University of Nizwa, Nizwa, Oman; 2 Oman Animal & Plant Genetic Resources Center, Muscat, Oman

**Keywords:** Lamiaceae, Chloroplast genomes, Phylogenetic analysis, Comparative analysis, *Teucrium* species

## Abstract

*Teucrium* is one of the most economically and ecologically important genera in the Lamiaceae family; however, it is currently the least well understood at the plastome level. In the current study, we sequenced the complete chloroplast (cp) genomes of *T. stocksianum* subsp.* stenophyllum* R.A.King (TSS), *T. stocksianum* subsp. *stocksianum* Boiss. (TS) and *T. mascatense* Boiss. (TM) through next-generation sequencing and compared them with the cp genomes of related species in Lamiaceae (*Ajuga reptans* L.,* Caryopteris mongholica* Bunge,* Lamium album* L.,* Lamium galeobdolon* (L.) Crantz, and *Stachys byzantina* K.Koch). The results revealed that the TSS, TS and TM cp genomes have sizes of 150,087, 150,076 and 150,499 bp, respectively. Similarly, the large single-copy (LSC) regions of TSS, TS and TM had sizes of 81,707, 81,682 and 82,075 bp, respectively. The gene contents and orders of these genomes were similar to those of other angiosperm species. However, various differences were observed at the inverted repeat (IR) junctions, and the extent of the IR expansion into ψ*rps19* was 58 bp, 23 bp and 61 bp in TSS, TS and TM, respectively. Similarly, in all genomes, the *pbsA* gene was present in the LSC at varying distances from the J_LA_ (IRa-LSC) junction. Furthermore, 89, 72, and 92 repeats were identified in the TSS, TM and TS cp genomes, respectively. The highest number of simple sequence repeats was found in TSS (128), followed by TS (127) and TM (121). Pairwise alignments of the TSS cp genome with related cp genomes showed a high degree of synteny. However, relatively lower sequence identity was observed when various coding regions were compared to those of related cp genomes. The average pairwise divergence among the complete cp genomes showed that TSS was more divergent from TM (0.018) than from TS (0.006). The current study provides valuable genomic insight into the genus *Teucrium* and its subspecies that may be applied to a more comprehensive study.

## Introduction

Lamiaceae is one of the largest families in the plant kingdom and comprises 240 genera and almost 72,000 species, which are distributed all over the world (Harley et al., 2004; Salmaki et al., 2016). The genus *Teucrium* consists of approximately 250 species (Harley et al., 2004) belonging to the family Lamiaceae, the second largest genus of subfamily *Ajugoideae* and are primarily perennial herbs, shrubs or subshrubs (Navarro & El Oualidi, 1999). The genus *Teucrium* contains medicinally important and essential-oil-rich plants (Miller & Morris, 1988; Ulubelen, Topu & Sönmez, 2000). *Teucrium* species have been used in medicines since ancient times, and many species of this genus possess important biological properties, such as antipyretic, anti-inflammatory, anti-ulcerogenic, antiseptic, anthelmintic, antitumour, hypoglycaemic, hypolipidaemic, and hepatoprotective antimicrobial activities (Abdollahi, Karimpour & Monsef-Esfehani, 2003; Barrachina et al., 1995; Sarac & Ugur, 2007). Two taxa of *Teucrium* (*T. stocksianum* subsp. *stenophyllum* and *T. mascatense*) are endemic to Oman (Ghazanfar, 1994). Furthermore, various taxa of *Teucrium* are found in the Arabian Peninsula and Middle East (Patzelt, 2015). Despite its considerable variation, this genus can be discriminated from closely related taxa by the combination of characteristics such as a 2-lipped to 5-lobed actinomorphic calyx, 1- (or rarely slightly 2-) lipped corolla, and arched or straight filaments (Salmaki et al., 2016). Furthermore, various factors, such as species richness, high phenotypic plasticity, ploidy variation and widespread distribution, play vital roles in the complexity of *Teucrium* and make it challenging and attractive for molecular phylogeneticists and systematists (Salmaki et al., 2016).

Chloroplast (cp) DNA is maternally inherited in the majority of angiosperm species but not in all (McCauley et al., 2007). Due to its mode of inheritance, cp DNA plays critical roles in molecular evolution and population genetic studies. Thus, cp DNA can be used not only for species discrimination but also to answer many other unsolved questions related to taxonomy (Liu et al., 2018a; McCauley et al., 2007; Reboud & Zeyl, 1994). Chloroplasts contain their own independent genomes and genetic systems, and DNA replication and transmission to daughter organelles result in the cytoplasmic inheritance of characteristics associated with the primary events in photosynthesis (Allen, 2015; Olmstead & Palmer, 1994). The cp genome is circular in structure, varies in size from 120 kb to 217 kb in angiosperms, and possesses a quadripartite configuration (Chumley et al., 2006; Wicke et al., 2011), being composed of a small single-copy (SSC) region and a large single-copy (LSC) region, which are generally separated by two copies of an inverted repeat region (IRa and IRb) (Wicke et al., 2011). Although the angiosperm cp genome is generally conserved in terms of gene order and gene content, in some angiosperm families, such as *Campanulaceae*, *Fabaceae*, *Geraniaceae*, and *Oleaceae*, the genome exhibits features such as gene, intron and even inverted repeat (IR) region loss, gene duplications, and large-scale rearrangements (Cai et al., 2008; Frailey et al., 2018; Greiner et al., 2008; Lee et al., 2007). Due to the conserved structure, recombination-free nature, and small size of the cp genome (Barrett et al., 2014), it is widely used in plant phylogenetic studies (Fan et al., 2018). The highly conserved structure of the cp genome facilitates primer design and sequencing, and cp DNA can be used as a barcode for plant identification (Shaw et al., 2005; Shaw et al., 2014).

With the advancement of genomic tools and methods, next-generation technologies have allowed the rapid sequencing of many cp genomes in recent years. These abundant cp genomes have facilitated the verification of evolutionary relationships and have allowed detailed phylogenetic classifications to be conducted at the group, family, and even genus levels in plants (Jansen et al., 2007; Parks, Cronn & Liston, 2009). Therefore, cp genome-scale data have increasingly been used to infer phylogenetic relationships at high taxonomic levels, and even at lower levels, great progress has been made (Barrett et al., 2013; Carbonell-Caballero et al., 2015; Moore et al., 2007; Shaw et al., 2014). Previously, many cp genomes had been sequenced and published from the Lamiaceae family, including *Ajuga reptans* L., *Caryopteris mongholica* Bunge (Liu et al., 2018b), *Lamium album* L., *Lamium galeobdolon* (L.) Crantz, and *Stachys byzantina* K.Koch (Liu et al., 2018b). In our current study, we sequenced the cp genomes of two subspecies of *T. stocksianum* (subspp. *stocksianum* and *stenophyllum*) and *T. mascatense* using a next-generation sequencing platform. These genomes are the first cp genomes to be reported from the genus *Teucrium.* Because these species possess morphological similarities in their habitats, in the current study, we aimed to sequence and determine the structures of the *Teucrium* cp genomes, to identify variations in simple sequence repeats (SSRs) and to identify repeat sequences in these eight cp genomes (TM, TS, TSS, *Ajuga reptans* L., *Caryopteris mongholica* Bunge, *Lamium album* L., *Lamium galeobdolon* (L.) Crantz, and *Stachys byzantina* K.Koch).

## Materials and Methods

### Sample collection

Young, fresh photosynthetic leaves from *Teucrium stocksianum* subsp. *stenophyllum* (TSS), *Teucrium stocksianum* subsp. *stocksianum* (TS) and *Teucrium mascatense* (TM) were collected from plants in Jabal Al Akhdar in Oman. The Director General of Nature Conservation from the Sultanate of Oman, Ministry of Environment & Climate Affairs issued the collection permit (4/2106). This sampling area is an arid land with a limited amount of rainfall and an average temperature of 25 °C; however, in the summer, temperatures can reach up to 33 °C, with a mean annual rainfall of 40 mm*.* The collected samples were washed with sterilized water, dried, placed immediately in liquid nitrogen and stored at −80 °C until cp DNA extraction. The specimens were deposited at the University of Nizwa Herbarium Center, Oman, with the voucher numbers UCTM11 (*Teucrium mascatense*), UCTS32 (*Teucrium stocksianum* subsp. *stenophyllum*), and UCTS30 (*Teucrium stocksianum* subsp. *stocksianum*).

### Chloroplast DNA extraction and sequencing

The leaves from TSS, TS and TM were ground into a fine powder in liquid nitrogen, and contamination-free cp DNA (nuclear- and mitochondrial-free DNA) was extracted according to a modified protocol including the addition of several purification steps (Shi et al., 2012). Genomic libraries were prepared according to the manufacturer’s instructions (Life Technologies, Carlsbad, CA, USA). The total cp DNA from each sample was sheared enzymatically into 400 bp fragments using the Ion Shear™ Plus Reagents kit, and libraries were prepared using the Ion Xpress™ Plus gDNA Fragment Library kit. Prepared libraries were quantified and qualified on a Qubit 3.0 fluorometer and bioanalyzer (Agilent 2100 Bioanalyzer system; Life Technologies, Carlsbad, CA, USA). Library preparation was followed by template amplification with the Ion OneTouch™ 2 instrument and the enrichment of the amplified template (Ion OneTouch™ ES enrichment system) using Ion 520 & 530 OT2 Reagents. The sample was loaded onto an Ion S5 Sequencing Chip, and sequencing was performed according to the protocol of the Ion Torrent S5.

### Genome assembly

A total of 1,246,225, 1,018,614 and 1,396,422 raw reads were generated for TSS, TS and TM, respectively. The obtained reads of the TSS, TS and TM genomes were mapped to the selected reference genome of *Ajuga reptans* (NC023102) using Bowtie2 (v.2.2.3) (Langmead & Salzberg, 2012) in Geneious Pro (v.10.2.3) (Kearse et al., 2012) software. The mean coverage of the assemblies for TSS, TS and TM were 186X, 128X and 256X, respectively. The IR junction regions were identified using the already published genome of *Ajuga reptans*, and an iteration method using MITObim (v.1.8) software (Hahn, Bachmann & Chevreux, 2013) was utilized to adjust the sequence length. After sequencing, FastQC (v0.11.6) (Andrews, 2015) was performed to check the read quality. To reduce biases in analysis, an in-house script was used to filter out reads if less than 90% of the bases that made up the read were below Q20. Trimmomatic (v0.36) (Bolger, Lohse & Usadel, 2014) was used to remove adapter sequences. Only high-quality reads were mapped using Bowtie2 in Geneious Pro (v.10.2.3) (Kearse et al., 2012).

### Genome annotation

The cp genomes were annotated with the Dual Organellar Genome Annotator (DOGMA) (Wyman, Jansen & Boore, 2004), BLASTX and BLASTN were used to identify the positions of ribosomal RNAs, tRNA and coding genes, and tRNAscan-SE version 1.21 (Schattner, Brooks & Lowe, 2005) software was used to annotate tRNA genes. Additionally, for manual adjustment, Geneious and tRNAscan-SE (Schattner, Brooks & Lowe, 2005) were used to compare the genomes with the previously reported *A. reptans* genome. Correspondingly, the start and stop codons and intron boundaries were also manually adjusted by comparison with the published *A. reptans* cp genome (NC_023102). In addition**,** the structural features of the *Teucrium* species cp genomes were illustrated using OGDRAW (Lohse, Drechsel & Bock, 2007). MEGA6 software (Kumar et al., 2008) was used to determine relative synonymous codon usage and deviations in synonymous codon usage while avoiding the influence of amino acid composition. The divergence of the genomes of these three *Teucrium* species from those of other related species was determined by using mVISTA (Frazer et al., 2004) in Shuffle-LAGAN mode, using *A. reptans* as the reference genome.

### Repeat identification

REPuter software (Kurtz et al., 2001) was used for the identification of palindromic and forward repeats in the genomes. The criterion was a minimum of 15 base pairs with sequence identities of 90%. Furthermore, simple sequence repeats (SSRs) were determined using Phobos version 3.3.12 (Kraemer et al., 2009), with the search parameters set as follows: for mononucleotide repeats, ≥10 repeat units; for dinucleotide repeats, ≥ 8 repeat units; for trinucleotide and tetranucleotide repeats, ≥4 repeat units; and for pentanucleotide and hexanucleotide repeats, ≥3 repeat units. Tandem Repeats Finder version 4.07 b (Benson, 1999), with the default settings, was used to determine tandem repeats.

### Sequence distance

The average pairwise sequence distance of the complete cp genomes and the genes shared among *Teucrium* species and other species were determined. Comparative sequence analyses were used to identify missing and ambiguous gene annotations after comparing gene orders and multiple sequence alignments. MAFFT version 7.222 (Katoh & Standley, 2013), with the default parameters, was used for the alignments of the complete cp genomes, and pairwise sequence distance was calculated using Kimura’s two-parameter (K2P) model (Kimura, 1980). A custom Python script (https://www.biostars.org/p/119214/) and DnaSP 5.10.01 (Librado & Rozas, 2009) were employed to determine single nucleotide polymorphisms (SNPs) and indel polymorphisms, respectively, among the complete genomes.

## Results

### Organization and general features of chloroplast genomes

**Figure 1:**
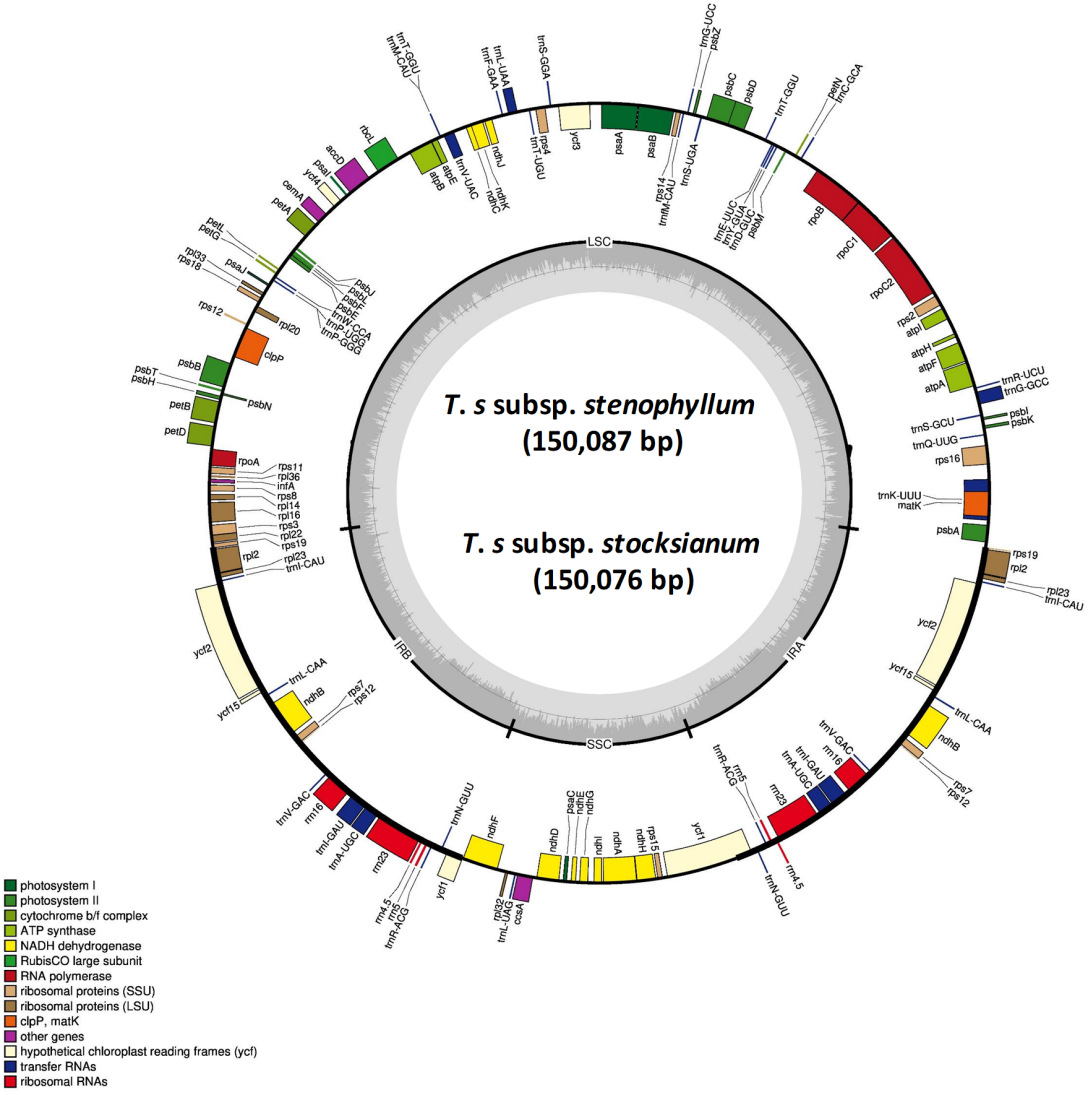
Genome map of the *T. stocksianum.* subsp. *stenophyllum* and *T. stocksianum* subsp. *stocksianum* cp genomes. Thick lines indicate the extent of the inverted repeat regions (IRa and IRb), which separate the genome into small (SSC) and large (LSC) single copy regions. Genes drawn inside the circle are transcribed clockwise, while those outside of the circle are transcribed counter clockwise. Genes belonging to different functional groups are colour coded. The dark grey in the inner circle corresponds to the GC content, while the light grey corresponds to the AT content.

The complete cp genomes of the three examined *Teucrium* species, *Teucrium mascatense* (TM), (MH325132; Data S1), *Teucrium stocksianum* subsp. *stenophyllum* (TSS) (MH325131; Data S2) and *Teucrium stocksianum* subsp. *stocksianum* (TS) (MH325133; Data S3) are circular molecules with quadripartite structures, similar to typical angiosperm cp genomes. The sizes of the TSS, TS and TM cp genomes are 150,087, 150,076 and 150,499 bp, respectively (Fig. 1 and Fig. S1). These cp genomes were compared with five related cp genomes with sizes ranging from 149,749 (*S. byzantina*) (Welch et al., 2016) to 151,707 bp (*C. mongholica*) (Table 1). The LSC regions of TSS, TS and TM are 81,707, 81,682 and 82,075 bp in length, respectively, while the sizes of the SSC regions are 17,182, 17,372 and 17,193 bp, respectively. The total numbers of genes annotated in these cp genomes are 135 in TSS and TS and 136 in TM, including 89 (TSS), 89 (TM), and 90 (TS) protein-coding genes, which accounted for 66,981, 66,487 and 67,100 bp in TSS, TM, and TS, respectively (Table 1). The total numbers of tRNAs in these genomes are 38 in TSS, 39 in TM and 37 in TS, and these numbers are similar to the numbers found in other cp genomes. The overall GC contents of the TSS, TS and TM genomes are 38.3%, 38.3% and 38.4%, respectively, and the highest GC content was observed in *S. byzantina* (38.7%) (Welch et al., 2016), while the lowest was 38.2% in *C. mongholica* (Liu et al., 2018b) (Table 1)*.* There are seventeen intron-containing genes in these three *Teucrium* species cp genomes, including three genes, *ycf3*, *clpP* and *rps12*, that contain two introns, while the remaining fourteen genes (*atpF*, *ndhA*, *ndhB*, *petB*, *petD*, *rpoC1*, *rpl2*, *rps16*, *trnA-UGC*, *trnG-GCC trnI-GAU*, *trnK-UUU*, *trnL-UAA*, and *trnV-UAC*) contain single introns, including eleven protein-coding genes (Table 2). The lengths of the introns vary among these genomes (Table 2). These genomes contain important genes responsible for the photosynthesis and self-replication of chloroplasts, as chloroplasts undergo independent replication (Table S1). These genes encode nine large ribosomal proteins, 12 small ribosomal proteins, 5 genes for photosystem I and 15 genes for photosystem II (Table S1). The total coding sequences in TM, TS and TSS were 66,487, 67,100 and 66,981 bp in length, including 21,190, 20,034 and 20,191 codons, respectively (Table 3).

**Table 1:** Summary of complete chloroplast genomes.

	***T. stenophyllum***	***T. mascatense***	***T. stocksianum***	***A. reptans***	***C. mongholica***	***L. album***	***L. galeobdolon***	***S. byzantina***
Size (bp)	150087	150499	150076	149963	151707	150505	151328	149749
Overall GC contents	38.3	38.3	38.3	38.3	38.2	38.6	38.5	38.7
LSC size in bp	81707	82075	81682	81769	83202	82444	82262	81270
SSC size in bp	17182	17193	17372	17102	17226	17177	17959	17679
IR size in bp	25599	25615	25511	25546	25639	25442	25553	25550
Protein coding regions size in bp	60573	56503	63572	78156	77946	80187	80400	80427
tRNA size in bp	2862	2961	2794	2842	2770	2793	2793	2794
rRNA size in bp	9050	9050	9048	9162	9162	9052	9052	9054
Number of genes	133	134	133	130	132	133	133	133
Number of protein coding genes	87	87	88	84	86	89	89	88
Number of rRNA	8	8	8	8	8	8	8	8
Number of tRNA	38	39	37	38	37	37	37	37
Genes with introns	14	14	14	15	15	14	15	15

**Notes.**

TM *Teucrium mascatense* TS *Teucrium stocksianum* subsp. *stocksianum* TSS *Teucrium stocksianum* subsp. *stenophyllum*

**Table 2:** The genes with introns in the Three *Teucrium* species chloroplast genome and the length of exons and introns.

**Gene**	**Location**	**Exon I (bp)**			**Intron 1 (bp)**			**Exon II (bp)**			**Intron II (bp)**			**Exon III (bp)**		
		TSS	TS	TM	TSS	TS	TM	TSS	TS	TM	TSS	TS	TM	TSS	TS	TMM
*atpF*	LSC	159	159	159	620	620	621	471	471	471						
*petB*	LSC	6	6	6	686	693	684	642	654	642						
*PetD*	LSC	9	9	9	701	701	695	525	525	525						
rpl2*	IR	393	393	393	676	676	661	435	435	435						
*rps16*	LSC	40	40	40	912	912	908	227	227	227						
*rpoC1*	LSC	456	456	456	803	805	826	1614	1614	1611						
rps12*	IR/LSC	114	114	114				232	232	232	539	539	539	26	26	26
*clpP*	LSC	69	69	69	732	732	732	291	291	294	626	626	626	228	228	228
*ndhA*	SSC	552	552	564	976	979	1028	540	540	540						
ndhB*	IR	777	777	777	679	679	680	756	756	756						
*ycf3*	LSC	129	129	129	708	708	708	228	228	228	731	737	736	153	153	153
trnA-UGC*	IR	38	38	38	806	806	813	35	35	35						
trnI-GAU*	IR	42	42	42	947	947	949	35	35	35						
*trnL-UAA*	LSC	37	37	37	480	480	480	50	50	50						
*trnK-UUU*	LSC	37	37	37	2480	2480	2480	26	26	26						
*trnG-GCC*	LSC	35			709			37								
*trnV-UAC*	LSC	38	38	38	578	578	576	37	37	37						

**Notes.**

TM *Teucrium mascatense* TS *Teucrium stocksianum* subsp. *stocksianum* TSS *Teucrium stocksianum* subsp. *stenophyllum*

**Table 3:** Base composition of the *Teucrium* chloroplast genome.

	**T/U**			**C**			**A**			**G**			**Length (bp)**		
	TSS	TS	TM	TSS	TS	TM	TSS	TS	TM	TSS	TS	TM	TSS	TS	TM
Genome	31.3	31.3	31.2	19.4	19.5	19.4	30.5	30.4	30.5	18.8	18.9	18.9	150087	150499	150076
LSC	32.6	32.6	32.5	18.6	18.7	18.6	31.0	30.9	31.0	17.8	17.8	17.8	81707	82075	81682
SSC	33.7	33.7	33.7	16.6	17.0	16.6	34.2	33.8	34.1	15.4	15.5	15.6	17182	17193	17372
IR	28.3	28.3	28.3	20.8	20.8	20.8	28.3	28.4	28.3	22.6	22.5	22.6	25599	25615	25511
tRNA	25.5	25.4	25.1	23.2	23.2	23.2	22.2	22.2	22.3	29.1	29.1	29.3	2862	2961	2794
rRNA	18.8	18.7	18.6	23.8	23.8	23.9	26.1	26.1	26.1	31.4	31.5	31.4	9050	9048	9048
Protein Coding genes	31.5	31.3	31.6	18	18.1	18	29.4	29.6	29.5	21.1	20.9	21.0	60073	56503	63572
1st position	22.92	25.84	21.052	18.96	19.89	16.80	29.52	21.32	26.91	28.59	31.98	24.10	20191	18834	21190
2nd position	33.45	37.71	32.62	21.10	22.02	18.28	26.98	30.42	24.09	18.45	20.78	16.63	20191	18834	21190
3rd position	36.35	42.95	29.87	15.03	15.77	13.24	31.62	35.78	27.91	16.19	17.98	15.092	20191	18834	21190

**Notes.**

TM *Teucrium mascatense* TS *Teucrium stocksianum* subsp. *stocksianum* TSS *Teucrium stocksianum* subsp. *stenophyllum*

### SSR analysis and repeats: an insight into the genome

**Figure 2:**
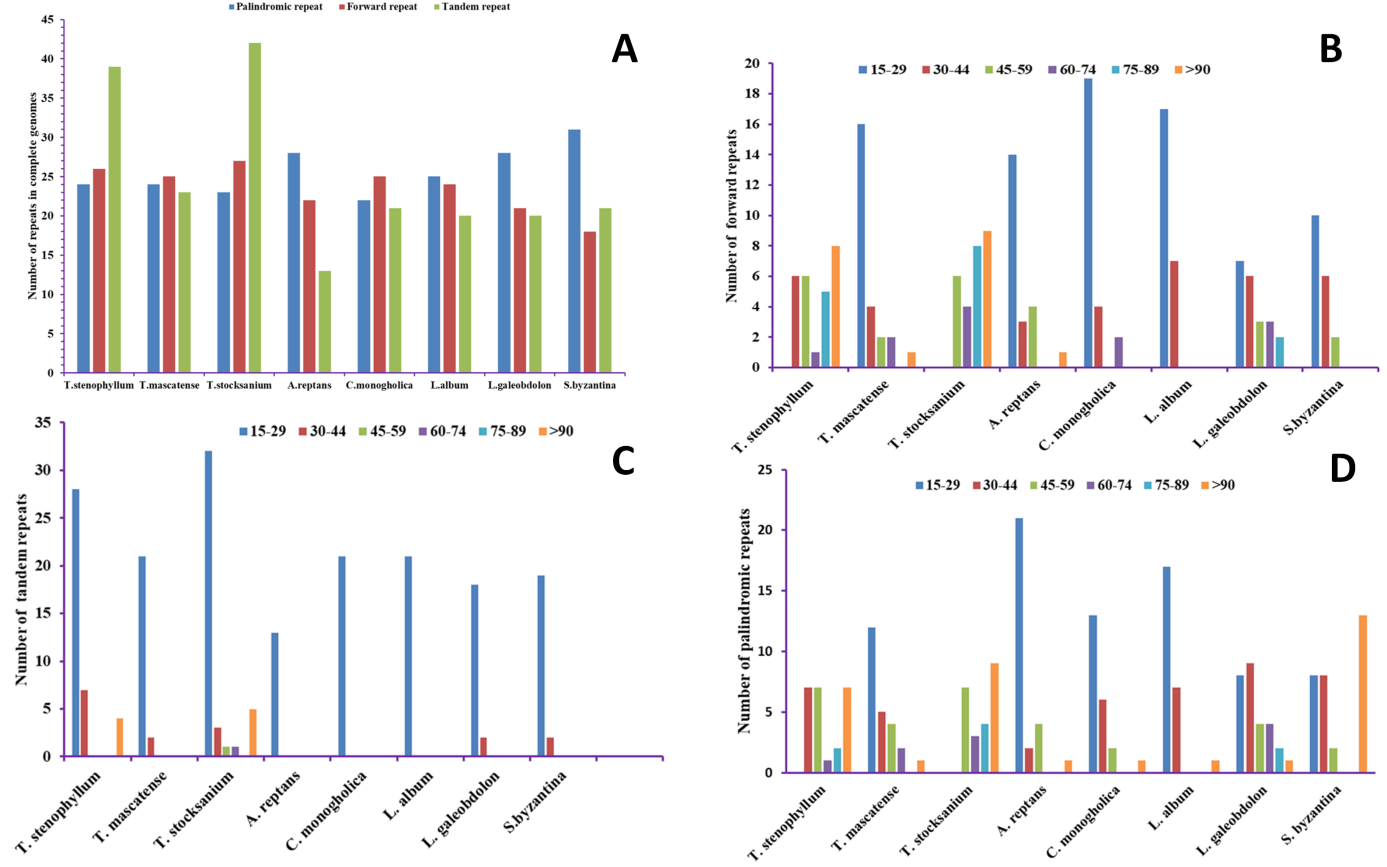
Analysis of repeated sequences in *T. mascatense, T. stocksianum* subsp. *stenophyllum* and *T. stocksianum* subsp. *stocksianum*. (A) Total numbers of the three repeat types, (B) frequencies of forward repeats by length, (C) frequencies of tandem repeats by length and (D) frequencies of palindromic repeats by length.

A total of 89, 72, and 92 repeats were found in the TSS, TM and TS cp genomes, respectively. The TSS cp genome contains 24 palindromic, 26 forward, and 39 tandem repeats; the TM cp genome contains 24 palindromic, 25 forward, and 23 tandem repeats; and the TS cp genome contains 23 palindromic, 27 forward and 42 tandem repeats (Fig. 2). The total numbers of repeats in the cp genomes of related species were also analysed, and 63, 68, 69, 69 and 70 total repeats were detected in the *A. reptans*, *C. mongholica* (Liu et al., 2018b), *L. album*, *L. galeobdolon* and *S. byzantina* cp genomes, respectively (Fig. 2). With 31 palindromic repeats, *S. byzantina* contains the highest number of palindromic repeats (Welch et al., 2016), while TSS contains the highest number of forward repeats at 26, and TS contains 39 tandem repeats, the highest among the compared genomes. We found that *C. mongholica* (Liu et al., 2018b) contains the lowest number of palindromic repeats (22), *S. byzantina* (Welch et al., 2016) contains the lowest number of forward repeats (18), and *A. reptans* contains the lowest number of tandem repeats (13) (Fig. 2).

**Figure 3:**
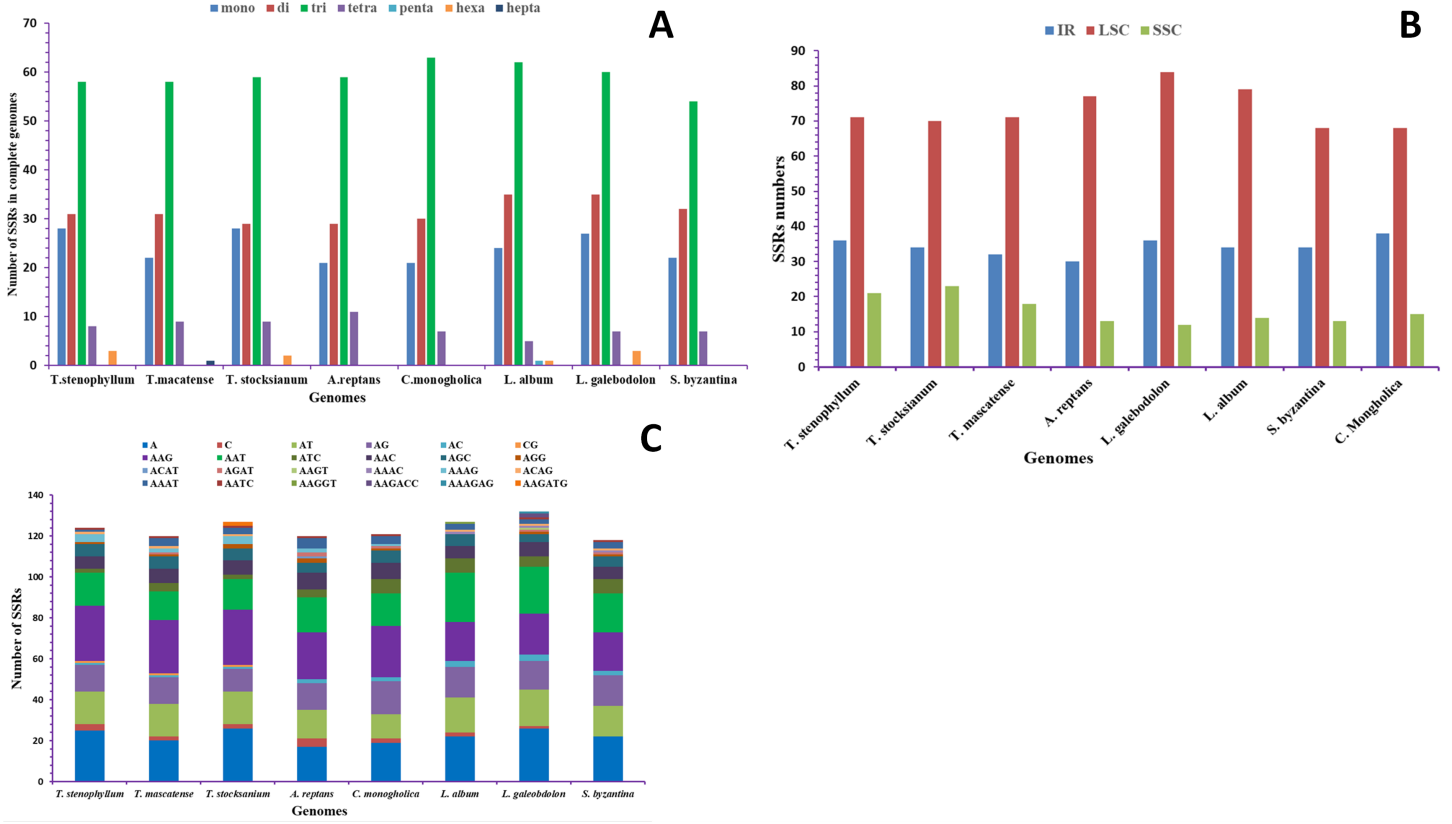
Analysis of simple sequence repeats (SSRs) in the *T. mascatense, T. stocksianum* subsp. *stenophyllum* and *T. stocksianum* subsp. *stocksianum* plastid genomes. (A) Number of SSR types in complete genome, (B) Number of SSR types in LSC, SSC and IR regions, and (C) frequency of identified SSR motifs in different repeat class types.

SSRs were also identified in the three *Teucrium* cp genomes and in five other genomes from the Lamiaceae family*.* The highest number of SSRs was found in TSS (128 SSRs), while the lowest number of SSRs was observed in *S. byzantina* (121 SSRs) (Welch et al., 2016). Trinucleotide repeats were found to be the most common type of SSRs, comprising 47.67% of all SSRs (Fig. 3). Most SSRs in TSS were trinucleotide repeats (58), followed by di- (31), mono- (28), tetra- (8) and hexanucleotide (3) repeats (Table S2). In TS, most SSRs were trinucleotide repeats (59), followed by di- (29), mono- (28), tetra- (9) and hexanucleotide (3) repeats (Table S3). In TM, the number of trinucleotide SSR repeats was 58, followed by 31 dinucleotide repeats, 28 mononucleotide repeats, 8 tetranucleotide repeats, 3 hexanucleotide repeats and 1 heptanucleotide repeat, which was found in only this genome (Fig. 3, Table S4). Interestingly, pentanucleotide SSRs were found in only the *L. album* cp genome.

### Characteristics of junctions in the cp genomes

**Figure 4:**
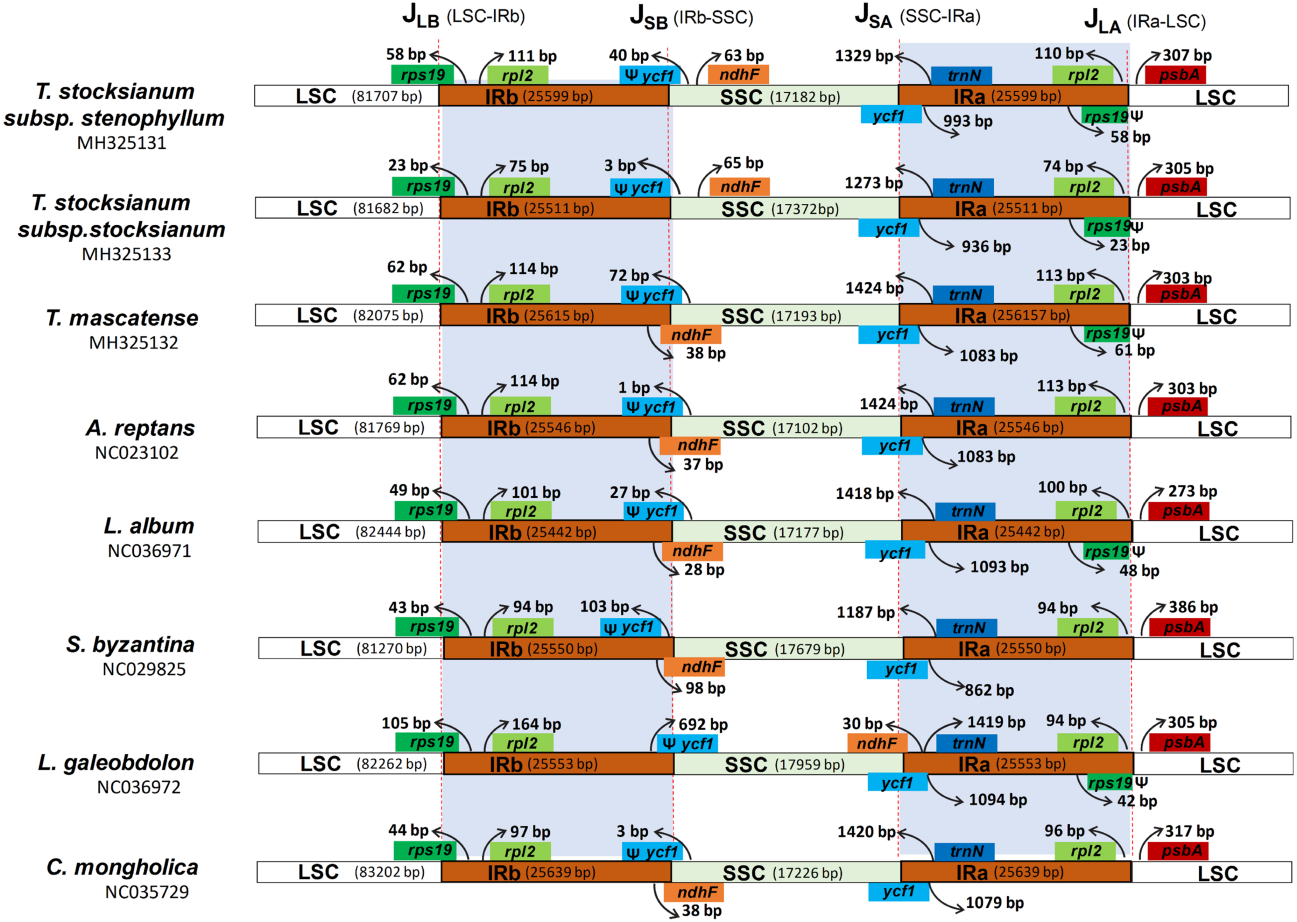
Distances between adjacent genes and junctions of the small single-copy (SSC), large single-copy (LSC), and two inverted repeat (IR) regions among eight plastid genomes within the family Lamiaceae. Boxes above and below the primary line indicate the adjacent border genes. The figure is not to scale with regards to sequence length and only shows relative changes at or near the IR/SC borders.

One of the aims of our study was to compare the actual positions of junctions within the three *Teucrium* cp genomes (TSS, TS and TM) and to compare these junction positions with those of three other cp genomes (*A. reptans*, *L. album*, and *S. byzantina*). The overall gene orientations, gene contents, and structures of these *Teucrium* species were the same, but these genomes possessed obvious differences at the junctions, similar to what has been observed in other typical cp genomes (Fig. 4). At J_LB_(LSC-IRb), the *rps19* gene is present, exceeding the IRb region by 58 bp in TSS, 23 bp in TS and 62 bp in TM. The *rpl2* gene is present in the IRb region in all genomes at varying distances from the junction. The J_SB_(IRb-SSC) junction is located in the ψ*ycf1* gene, a pseudogene in the IRb region with a length equivalent to the length that the IRa is expanded into the ψ*ycf1* gene. Interestingly, the *ndhF* gene is present in the SSC regions of both TSS and TS, while in TM, it is present at the J_SB_ junction, overlapping the ψ*ycf1* gene (Fig. 4). Moreover, the J_LA_(IRa-LSC) border is characteristically located upstream of ψ*rps19* and downstream of the *psbA* gene. The IRa region was expanded to partially include ψ*rps19*, creating a truncated ψ*rps19* copy at the J_LA_ border in all the examined *Teucrium* species. However, in *S. byzantina*, this pseudogene is missing. The extent of the IR expansion into ψr*ps19* is 58 bp, 23 bp and 61 bp in TSS, TS and TM, respectively. Similarly, in all genomes, the *pbsA* gene is present in the LSC at varying distances from the J_LA_junction.

### Comparative analysis of sequence variation

**Figure 5:**
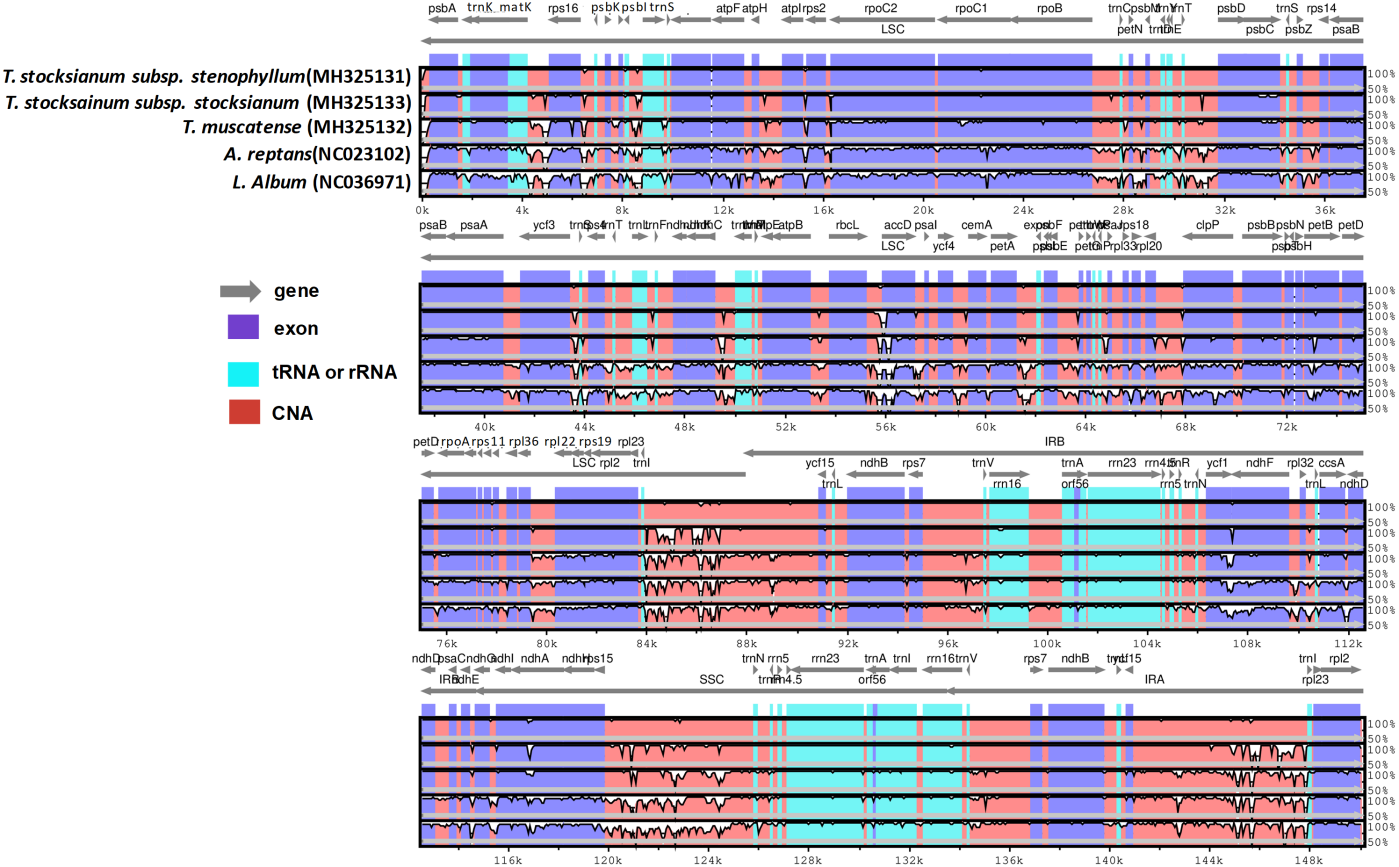
Visual alignment of plastid genomes from *T. mascatense, T. stocksianum* subsp. *stenophyllum* and *T. stocksianum* subsp. *stocksianum* with previously reported A. reptans and L. album cp genomes. VISTA-based identity plot showing sequence identity among eight species, using *T. stocksianum* subsp. *stenophyllum* as a reference.

Comparisons among these genomes using mVISTA revealed several regions of sequence variation. The TSS genome was used as the reference genome. Some genes, such as *rps16*, *rpoC1*, *ycf3*, *accD*, *clpP*, *petB*, *petD*, *accD*, *ycf1*, *ndhA*, *petD*, and *atpF*, showed sequence variation among these genomes. In the IRb region, the most divergent regions among the compared genomes was the *rps7*-*trnV* region. In the LSC region, the *rpoC1* gene showed sequence variation only in *A. reptans* and *L. album*, as did the *rps16* and *petB* genes. In the SSC region, the *ndhA* gene also showed sequence divergence among the compared genomes (Fig. 5).

Following these findings, we calculated the average pairwise sequence distances among the complete cp genomes of these eight species (*A. reptans*, *C. mongholica*, *L. album*, *L. galeobdolon*, *S. byzantina*, TM, TS, and TSS), and the results revealed that TSS had greater sequence divergence from TM (0.018) than from TS (0.006). We found that the genome sequence distances between TS and TM were smaller than those when these genomes were compared to other genomes (Table S5). Furthermore, when the TSS genome was compared with those of TM, TS, *A. reptans*, *C. mongholica*, *L. album*, *L. galeobdolon* and *S. byzantina* with respect to indels and SNPs, 5,458, 6,254, 10,130, 14,106, 19,575, 18,982, and 24,008 SNPs were detected, while 2,275, 2,275, 13,291, 12,991, 18,241, 18,201, and 29,438 indels were detected, respectively (Table S6).

## Discussion

The genomic structures and gene orders of the *Teucrium* cp genomes are highly conserved, and no rearrangement has occurred. The IRs of the *Teucrium* species are approximately 25.5 kb in length, and this value is within the size range found in most angiosperm cp genomes (20 ± 28 kb) (Chumley et al., 2006). The TSS cp genome is 150,087 bp, the TS genome is 150,076 bp, and the TM genome is 150,499 bp, and the sizes of these reported cp genomes are consistent with the sizes of the previously reported cp genomes of *C. mongholica* (151,707 bp) (Liu et al., 2018b), *Salvia miltiorrhiza* (151,328 bp) (Qian et al., 2013), *Origanum vulgare* L. (151,935 bp) and *Mentha spicata* (152,132 bp) (Lukas & Novak, 2013)*.* The *Teucrium* cp genome has a typical quadripartite structure and consists of an SSC and an LSC separated by a pair of IRs. All the sequenced *Teucrium* species contain higher AT content than GC content. The GC content in TSS, TS, TM and *A. reptans* is almost identical, and similarly, the GC content in *C. mongholica* is 38.2% (Abu-Irmaileh & Afifi, 2003), that in *L. album* is 38.6%, that in *L. galeobdolon* is 38.5% and that in *S. byzantina* is 38.7%. The IR region has a higher GC content than the non-coding intergenic regions due to the presence of rRNA genes (Bock, 2007).

The gene orders and gene contents of these genomes are conserved. The number of genes in these *Teucrium* cp genomes are similar to the numbers that were previously reported in the *M. spicata* (Wang et al., 2017), *Lavandula angustifolia* (Ma, 2018), and *Perilla frutescens* (L.) Britton (Shen et al., 2016) genomes. The number of intron-containing genes (14) in the sequenced genomes (TM, TS, and TSS) was similar in this study. With the exception of the *A. reptans* and *C. mongholica* genomes (Liu et al., 2018b), which contain 15 intron-containing genes, the intron contents of cp genomes are conserved; however, in some species such as *Lagerstroemia fauriei*, structural changes, such as sequence losses or variations (SNP), have been reported (Gu et al., 2016). Some genes, such as *atpF* (ATP synthase), *rpoC2* (RNA polymerase) and ribosomal proteins (*rpl12*, *rps12*, and *rps16*), are known to have structural intron variation (Daniell et al., 2016; He et al., 2017). The cp genome can gain or lose introns during evolution, and this process plays an important role in the regulation of gene expression through the stabilization of the transcript or through alternative splicing (Daniell et al., 2008). Our results reveal that there are 11 protein-coding genes, six tRNA genes (TSS) and five tRNA genes (TS and TM) that contain introns. As in the previously reported cp genomes, both *clpP* and *ycf3* contain double introns. The previously reported *O. vulgare* cp genome (Lukas & Novak, 2013) shows a similar result, while in the *S. miltiorrhiza* (Qian et al., 2013) cp genome, there are nine protein-coding genes and six tRNAs that contain introns, and the number of double-intron-containing genes is three (Qian et al., 2013).

In most land plants, the cp genome has a collinear gene order, but it also displays some remarkable changes, such as sequence inversion (Cho et al., 2015), gene loss (Fu et al., 2016), and expansion and contraction at the borders between the LSC, the SSC and the IRs (Choi, Chung & Park, 2016). The expansion and contraction of the IR regions often results in the length variations observed among cp genomes (Cho & Park, 2016; Hu, Woeste & Zhao, 2017). In some genomes, such as Fabaceae (Wang et al., 2018) *Erodium* and *Sarcocaulon* (Blazier et al., 2016), the loss of IRs has also been reported. The differences in genome size among the sequenced and compared species can be explained by the variations in the LSC, SSC and IR regions. The sizes of the cp genomes of the three *Teucrium* species (TSS, TS, and TM) differ, and there are some notable variations in the junction regions. The boundaries between the LSC, the SSC and the IRs were identical in all the cp genomes studied. The LSC/IRb boundary of the three *Teucrium* cp genomes and of the compared genomes is located in the *rps19* gene, and a small fraction of the *rps19* gene is also located in the IRb region, similar to the previously reported *S. miltiorrhiza* cp genome (Qian et al., 2013) and *O. vulgare* cp genome (Lukas & Novak, 2013) and the cp genomes from seven species from the genus *Ilex* (Yao et al., 2016). In contrast, there are some cp genomes in which the *rps19* gene does not extend into the IR region, such as the *Millettia pinnata* cp genome (Kazakoff et al., 2012) and *Lupinus luteus* cp genome (Martin et al., 2014). It has been reported in various studies (Wang et al., 2008) that, mostly in monocots, the *rps19* gene occurs inside the IR region, as in the *Oryza* AA genome (Wambugu et al., 2015). The *ycf1* gene extends over the SSC/IRb junction and overlaps with the *ndhF* gene in most of the compared genomes, including TM, while in TSS and TS, the *ycf1* gene does not overlap with the *ndhF* gene and is located on both sides of the SSC and IRb; a similar result has also been observed in the *Petroselinum crispum*, *Tiedemania filiformis* and *Panax ginseng* cp genomes (Kim & Lee, 2004; Li et al., 2018).

Repetitive sequences, such as tandem repeats and SSRs, play important roles in the stabilization and rearrangement of cp genome sequences (Do Nascimento Vieira et al., 2014) and can affect copy number variation among different and similar species. Such features in cp genomes can be utilized for molecular marker design, which helps in plant identification at the molecular level (Cho et al., 2016) and phylogenetic analyses (Williams et al., 2016). We found that there are more repeats in the intergenic spacer regions than in the coding regions, as expected. Tandem repeats and SSRs can account for recombination in cp genomes, which leads to differences between genomes (Ogihara, Terachi & Sasakuma, 1988). The *Teucrium* species possess high numbers of repeats in their cp genomes, and it is evident from previous studies that large and complex repeats also play major roles in the rearrangement of sequences within cp genomes and in the evolution of cp genomes (Milligan, Hampton & Palmer, 1989; Cavalier-Smith, 2002; Bausher et al., 2006). Our findings show that TS has the highest number of repeats (92), while TSS has the lowest (53) number of repeats.

In our study, tandem repeats were found to be the most abundant in the *Teucrium* species genomes, showing similar traits to the previously reported *S. miltiorrhiza* cp genome (Qian et al., 2013). SSRs in the *Teucrium* species genomes primarily contain numerous AT subunits, with mononucleotide repeats comprising only A and T repeats. These results are consistent with those for the previously reported cp genomes of angiosperms (Qian et al., 2013; Khan et al., 2017), which have high AT contents (Nie et al., 2012). Furthermore, trinucleotide repeats were more abundant than any other type of nucleotide repeats in these studied genomes, and this finding is consistent with the findings of the previously reported cp genome of *Origanum vulgare* (Lukas & Novak, 2013).

Our study establishes that a higher level of variation was observed in the following regions of the three *Teucrium* species and two other compared species: *rps16*, *rpoC1*, *ycf3*, *accD*, *clpP*, *petB*, *petD*, *ycf1*, *ndhA*, and *atpF.* Therefore, these regions within the genus *Teucrium* are useful regions for elucidating phylogenetic relationships. These regions contain variation and are suitable for phylogenetic analysis of *Teucrium* and for evaluation of unresolved phylogenetic relationships. *Ycf1*, *ycf2*, *rpoC2*, and *ndhF* were confirmed to be the most divergent regions in the previously reported *S. miltiorrhiza* cp genome within the Lamiaceae family (Qian et al., 2013). Genes such as *rpoC1* and *ycf1* were also found to be among the most divergent in the six reported cp genomes from Asteraceae species (Nie et al., 2012). Moreover, coding regions such as *ndhA*, *rps16*, *accD*, *clpP*, *ccsA*, *infA*, *rpl22*, *rpl32* and *ycf1* were also found to be the most divergent genes in vascular plant cp genomes (Kumar et al., 2009).

## Conclusion

This study successfully mapped the first three cp genomes of the genus *Teucrium* from the family Lamiaceae using next-generation sequencing technology. The genome organizations and gene orders of these three *Teucrium* cp genomes demonstrated similarity among these three specimens as well as when compared to other genomes, such as *S. miltiorrhiza*, from the Lamiaceae family. Repetitive sequences, such as SSRs and tandem repeats, were determined within the eight cp genomes. Contraction and expansion as well as sequence divergence inside these genomes were also ascertained. The findings of our study will further facilitate the biological study of this medicinally significant plant genus.

##  Supplemental Information

10.7717/peerj.7260/supp-1 Table S1 Genes in the sequenced *T. mascatense, T. stocksianum subsp. stenophyllum* and *T. stocksianum* subsp. *stocksianum* genome

10.7717/peerj.7260/supp-2 Table S2 Base compositions in *T. mascatense, T. stocksianum subsp. stenophyllum* and *T. stocksianum* subsp. *stocksianum* genome cp genomes

10.7717/peerj.7260/supp-3 Table S3 Simple sequence repeats (SSRs) in *T. mascatense* chloroplast genome

10.7717/peerj.7260/supp-4 Table S4 Simple sequence repeats (SSRs) in *T. Stocksianum* subsp.* stocksianum* chloroplast genome

10.7717/peerj.7260/supp-5 Table S5 Simple sequence repeats (SSRs) in *T. stocksianum* subsp. *Stenophyllum* chloroplast genome

10.7717/peerj.7260/supp-6 Table S6 Pairwise distance of *Teucrium* species cp genome with related species cp genomes

10.7717/peerj.7260/supp-7 Figure S1 Genome map of the *T. mascatense* Thick lines indicate the extent of the inverted repeat regions (IRa and IRb), which separate the genome into small (SSC) and large (LSC) single copy regions. Genes drawn inside the circle are transcribed clockwise, and those outside are transcribed counter clockwise. Genes belonging to different functional groups are color-coded. The dark grey in the inner circle corresponds to the GC content and the light grey corresponds to the AT content.

10.7717/peerj.7260/supp-8 Data S1 Raw GenBank file MH325132

10.7717/peerj.7260/supp-9 Data S2 RAW GenBank file MH325131

10.7717/peerj.7260/supp-10 Data S3 RAW GenBank file MH325233

## Additional Information and Declarations

### Field Study Permissions

The following information was supplied relating to field study approvals (i.e., approving body and any reference numbers): The Director General of Nature Conversation from the Sultanate of Oman, Ministry of Environment & Climate Affairs provided a permit to collect wild plant specimens (permit no. 4/2106).

### DNA Deposition

The following information was supplied regarding the deposition of DNA sequences: Sequences are available at GenBank with accession numbers: MH325131, MH325132, and MH325133.

### Data Availability

The following information was supplied regarding data availability: The specimens are accessible at the University of Nizwa Herbarium Center, Oman, with the voucher numbers UCTM11 (*Teucrium mascatense*), UCTS32 (*Teucrium stocksianum* subsp. *stenophyllum*), and UCTS30 (*Teucrium stocksianum* subsp. *stocksianum*). http://sweetgum.nybg.org/science/ih/herbarium-list/?NamOrganisationAcronym=NMSRC.
