# Supplementary material for: Complete chloroplast genomes of medicinally important Teucrium species and comparative analyses with related species from Lamiaceae
Source: PeerJ. 2019 Jul 9;7:e7260. doi: 10.7717/peerj.7260 (PMC6625504; doi:10.7717/peerj.7260)
Supplement: Table S1 [file peerj-07-7260-s001.docx]

**Table S1. Genes in the sequenced *Teucrium* species chloroplast genomes.**

| **Category** | **Group of genes** | **Ontology source** | **Name of genes** |
| --- | --- | --- | --- |
| **Self-replication** | Large subunit of ribosomal proteins | *Cellular component* | *rpl2*, *14*, *16*, *20*, *22*, *23*, *32*, *33*, *36* |
|  | Small subunit of ribosomal proteins | *Cellular component* | *rps2, 3, 4, 7, 8, 11, 12, 14,15 ,16, 18, 19* |
|  | DNA dependent RNA polymerase | *Biological process/Molecular function* | *rpoA, B, C1, C2* |
|  | rRNA genes | *Biological process* | *rrn 4.5, rrn 5, rrn 16, rrn23* |
|  | tRNA genes | *Biological process* | *trnA-UGC, trnC-GCA, trnD-GUC, trnE-UUC trnF-GAA, trnfM-CAU, trnG-UCC, trnH-GUG, trnI-CAU, trnI-GAU, trnK-UUU, trnL-CAA, trnL-UAA, trnL-UAG, trnM-CAU, trnN-GUU, trnP-GGG, trnP-UGG, trnQ-UUG, trnR-ACG, trnR-UCU, trnS-GCU, trnS-GGA, trnS-UGA, trnT-GGU, trnT-UGU, trnV-GAC, trnV-UAC, trnW-CCA, trnY-GUA* |
| **Photosynthesis** | Photosystem I | *Cellular component* | *psaA, B, C, I, J,* |
|  | Photosystem II | *Cellular component* | *psbA, B, C, D, E, F, H, I, J, K, L, M, N, T, Z* |
|  | Cytochrome b6/f complex | *Molecular function* | *petA, B, D, G, L, N* |
|  | ATP synthase | *Molecular function* | *atpA, B, E, F, H, I* |
|  | Rubisco | *Cellular component* | *rbcL* |
| **Other genes** | Maturase | *Cellular component* | *matK* |
|  | Protease | *Cellular component* | *clpP* |
|  | Envelop membrane protein | *Molecular function* | *cemA* |
|  | Subunit Acetyl- CoA-Carboxylate | *Molecular function, Biological process* | *accD* |
|  | c-type cytochrome synthesis gene | *Biological process* | *ccsA* |
| **Unknown** | Conserved Open reading frames | *Cellular component (Ycf2)* | *Ycf1,2, 3,4, 15* |
