# Supplementary material for: Complete chloroplast genomes of medicinally important Teucrium species and comparative analyses with related species from Lamiaceae
Source: PeerJ. 2019 Jul 9;7:e7260. doi: 10.7717/peerj.7260 (PMC6625504; doi:10.7717/peerj.7260)
Supplement: Table S2 [file peerj-07-7260-s002.docx]

**Table S2. Simple sequence repeats (SSRs) in the chloroplast genome of *Teucrium stocksianum* subsp. *stenophyllum.***

| **Unit** | **Length** | **No** | **SSR start** |
| --- | --- | --- | --- |
| \| **A** \| \| --- \| \|  \| | 17 | 1 | 77741 |
|  | 12 | 3 | 99938,123817,131811 |
|  | 11 | 22 | 18364, 42355, 43565, 57645, 82208, 111948, 116962, 149542, 4499, 7940, 8186, 27142, 43676, 53121, 68383, 78260, 79450, 81682, 111847, 11687, 121640, 122630 |
| **C** | 11 | 2 | 101154, 130596 |
| **AT** | 10 | 1 | 45050 |
|  | 9 | 4 | 30326 53650 75747 120080 |
|  | 8 | 8 | 60231, 74022, 74238, 30318, 30335, 41425, 43598, 46708 |
| **AG** | 8 | 2 | 54335 59316 |
|  | 8 | 6 | 83944, 92312, 103299, 128454, 139441, 147809 |
|  |  |  |  |
| **AC** | 8 | 1 | 13837 |
| **CG** | 8 | 1 | 19727 |
|  |  |  |  |
| **AAAG** | 13 | 4 | 4335, 4433, 69887, 79880 |
| **ACAG** | 12 | 1 | 11088 |
| **AAAT** | 12 | 3 | 47111, 67747, 112167 |
| **AATC** | 12 | 1 | 119837 |
| **AAG** | 15 | 2 | 107122 124624 |
|  | 11 | 1 | 71589 |
|  | 10 | 1 | 28018 |
| **AAT** | 10 | 5 | 81813, 94045, 107074, 111868, 116453 |
|  | 10 | 3 | 124677 137706 149938 |
|  | 9 | 15 | 2926, 21984, 28706, 36159, 75922, 87896, 89641, 92607, 109428, 120788, 120951, 123277, 139145, 142111, 143856 |
