# Supplementary material for: Complete chloroplast genomes of medicinally important Teucrium species and comparative analyses with related species from Lamiaceae
Source: PeerJ. 2019 Jul 9;7:e7260. doi: 10.7717/peerj.7260 (PMC6625504; doi:10.7717/peerj.7260)
Supplement: Table S4 [file peerj-07-7260-s004.docx]

**Table S4. Simple sequence repeats (SSRs) in the chloroplast genome of *Teucrium mascatense.***

| Unit | Length | No | SSR start |
| --- | --- | --- | --- |
| A | 17 | 1 | 78166 |
|  | 16 | 1 | 8171 |
|  | 13 | 2 | 100291, 132273 |
|  | 12 | 2 | 124287, 65526 |
|  | 11 | 5 | 11637 43626 58071 68832 78686 |
|  | 10 | 9 | 7523 43574 53051 53133 53161 79875 112333 122084 122876 |
| C | 11 | 2 | 97825, 134741 |
| AT | 10 | 2 | 19737 45111 |
|  | 9 | 4 | 30369,53688 76171 120518 |
|  | 8 | 10 | 30361, 30378, 41485, 46770  60655, 74458, 74674,81753, 90860, 141709 |
| AG | 9 | 3 | 34615, 40231, |
|  | 8 | 10 | 54373, 59741, 84356, 85270, 92678, 103650, 128919, 139891, 147299,148213 |
| AC | 8 | 1 | 13831 |
| CG | 8 | 1 | 19727 |
| AAAG | 13 | 2 | 70337, 80304 |
| AGAT | 12 | 1 | 4757 |
| AAAT | 12 | 4 | 4998, 47174, 68199, 112551 |
| ACAG | 12 | 1 | 11069 |
| AATC | 12 | 1 | 120275 |
| AAT | 11 | 2 | 30572 65718 |
|  | 10 | 6 | \| 7582, \| 50171, \| 53105 \| 120200, \| 8560, \| 45105 \| \| --- \| --- \| --- \| --- \| --- \| --- \| |
|  | 9 | 3 | 57265 63800 110715 |
| AAG | 11 | 1 | 72038 |
|  | 10 | 22 | 17042, 28044, 82245, 94412,112253,116830,138155, 150322, 2922, 21988, 28738, 36214, 76348, 90017, 92973, 109805, 121224, 121370, 123739, 139595,142551,144302 144408 |
| ATC | 10 | 2 | 37746, 53541 |
|  | 9 | 2 | 88757, 143811 |
| AAC | 10 | 1 | 53856, 8627, 14608 |
|  | 9 |  | 48796, 66428, 107088, 125480 |
| AGC | 9 | 6 | 39647, 55268, 81616, 102083, 118089, 130485 |
| AGG | 9 | 1 | 54544 |
