# Supplementary figures and images for: Complete chloroplast genomes of medicinally important Teucrium species and comparative analyses with related species from Lamiaceae
Source: PeerJ. 2019 Jul 9;7:e7260. doi: 10.7717/peerj.7260 (PMC6625504; doi:10.7717/peerj.7260)

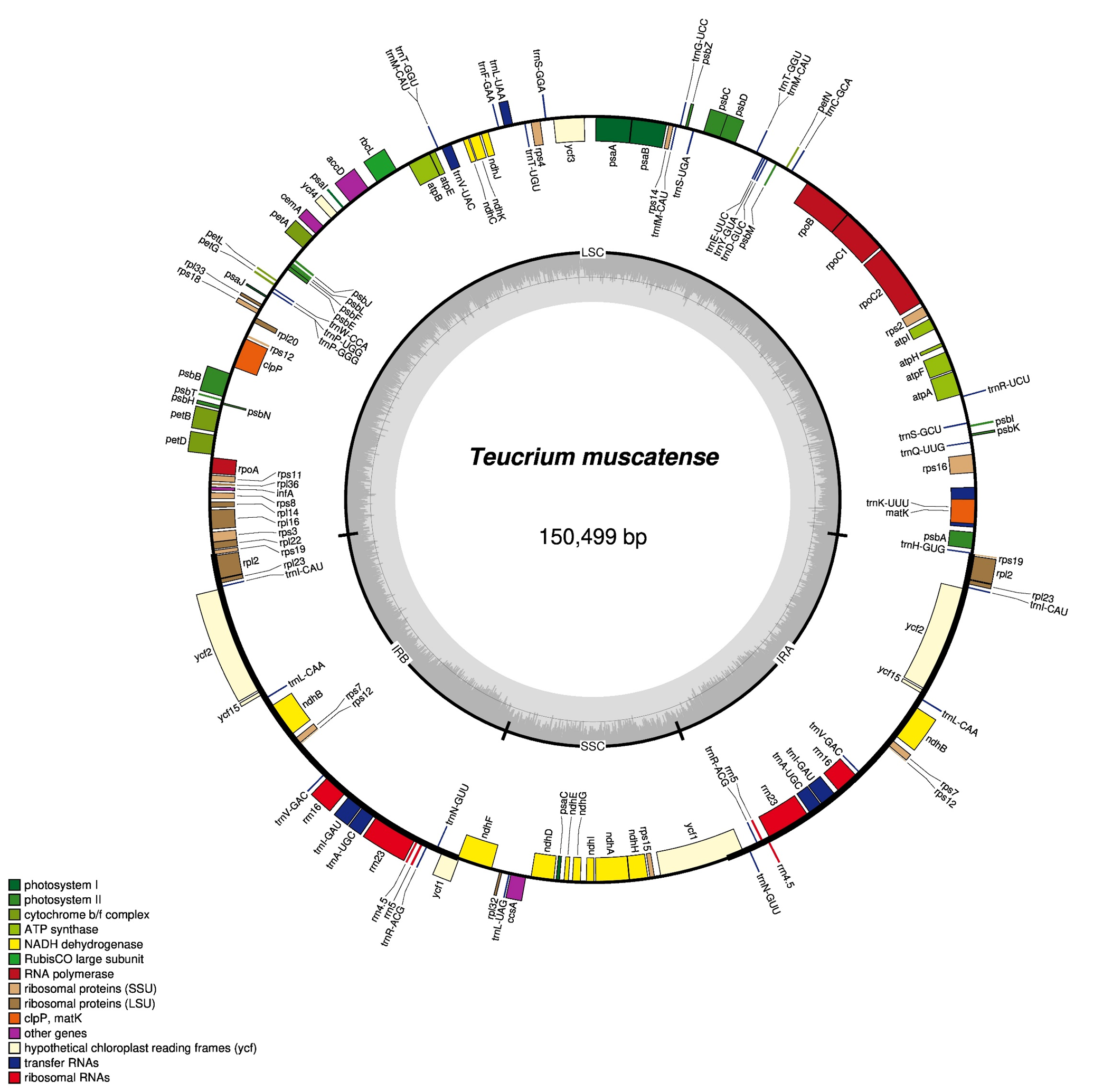

Supplement: Figure S1 — Thick lines indicate the extent of the inverted repeat regions (IRa and IRb), which separate the genome into small (SSC) and large (LSC) single copy regions. Genes drawn inside the circle are transcribed clockwise, and those outside are transcribed counter clockwise. Genes belonging to different functional groups are color-coded. The dark grey in the inner circle corresponds to the GC content and the light grey corresponds to the AT content. [file peerj-07-7260-s007.png]
